# A case of Schnitzler’s syndrome without monoclonal gammopathy successfully treated with canakinumab

**DOI:** 10.1186/s12891-021-04120-z

**Published:** 2021-03-08

**Authors:** Yuya Fujita, Tomoyuki Asano, Akira Sakai, Natsumi Norikawa, Toshiyuki Yamamoto, Haruki Matsumoto, Shuzo Sato, Jumpei Temmoku, Makiko Yashiro-Furuya, Naoki Matsuoka, Hiroshi Watanabe, Kiyoshi Migita

**Affiliations:** 1 Department of Rheumatology, Fukushima Medical University School of Medicine, 1 Hikarigaoka, Fukushima, Fukushima 960-1295 Japan; 2 Department of Radiation Life Sciences, Fukushima Medical University School of Medicine, 1 Hikarigaoka, Fukushima, Fukushima 960-1295 Japan; 3 Department of Dermatology, Fukushima Medical University School of Medicine, 1 Hikarigaoka, Fukushima, Fukushima 960-1295 Japan

**Keywords:** Autoinflammatory disease, Canakinumab, Interleukin-1, PET/CT, Schnitzler’s syndrome, Urticaria

## Abstract

**Background:**

Schnitzler’s syndrome (SchS) is a rare autoinflammatory syndrome with diagnostic challenge and be characterized by chronic urticaria, a monoclonal gammopath, periodic fever and bone pain. In addition to the monoclonal gammopathy, bone abnormalities are often found at the site of bone pain in patients with SchS. The remarkable efficacy of interleukin-1 (IL-1) inhibition was also demonstrated in this syndrome.

**Case presentation:**

We describe a case of refractory chronic urticaria presenting with clinical manifestations consistent with SchS without monoclonal gammopathy. A 43-year-old female patient suffering from recurring of urticaria with periodic fever as well as bone pain for the past 4 years. The patient had leukocytosis and elevated levels of C-reactive protein (CRP) and serum amyloid A (SAA). PET/CT (positron emission tomography/computed tomography) and MRI (magnetic resonance imaging) examination revealed hyper-metabolism areas in both femoral bone marrow. Although bone marrow histology revealed no abnormality, urticarial skin lesions shows neutrophilic infiltrations without evidence of vasculitis. We could not exclude the possibility of SchS. The patient had been treated with antihistamines, steroids, omarizumab, colchicine and cyclosporine A, no therapeutic effect was observed. She was started on canakinumab 150 mg subcutaneous injection with 4 weeks interval. Within 48 h after the first injection, the urticarial rash disappeared, and febrile attack and bone pain had not recurred. Elevated levels of serum CRP and SAA were normalized within a week after the first injection of canakinumab.

**Conclusions:**

The current case suggests an important role for IL-1 as a mediator in the pathophysiology of SchS-like refractory urticaria with bine pain. It had been presumed that monoclonal gammopathy may not always present in SchS. It is important to avoid delay in diagnosis and initiation of proper treatment in SchS or autoinflammatory conditions resembling SchS.

## Background

Chronic urticaria may be associated with underlying diseases, including autoinflammatory diseases [1]. Schnitzler’s syndrome (SchS) is a rare and under-recognized syndrome characterized by chronic urticaria, a monoclonal gammopath, periodic fever; bone pain; and elevated levels of acute phase reactants [2]. The diagnostic criteria for this disorder include recurrent and nonprutic urticaria and monoclonal gammopathy (IgM Kappa light chain, > 90%) [3]. Additionally, the following minor criteria required, recurrent fever, objective finding of abnormal bone remodeling with bone pain (assessed by bone scintigraphy, or MRI), neutrophilic dermal infiltrate on skin biopsy and elevated CRP and/or leukocytosis (CRP of > 30 mg/L, and/or neutrophils of > 10,000/mm^3^) [4]. Although the etiology of this syndrome is unknown, recent evidence suggest it categorized as an autoinflammatory disease [5]. Recognition of this disease is critical, since it could be associated with a serious complication, amyloid A (AA) amyloidosis [6]. Here, we report a refractory case of urticarial rash accompanied with longstanding recurrent febrile attack and bone pain with similarity to SchS without monoclonal gammopathy successfully treated with canakinumab.

## Case presentation

**Fig. 1:**
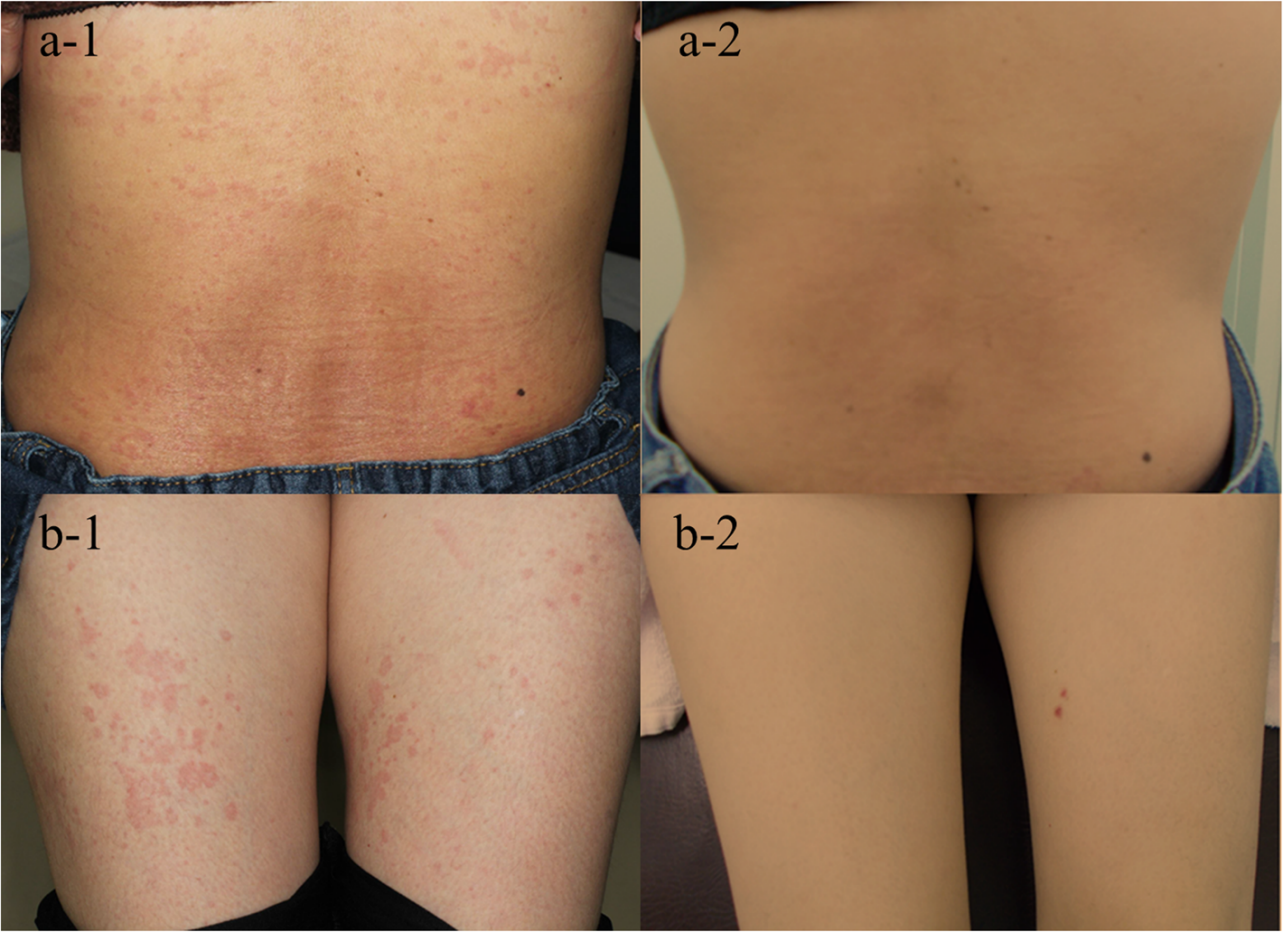
Cutaneous manifestation. Cutaneous manifestation at diagnosis. The urticarial recurrent rash was shown in body trunk (**a-1**) and femurs (**b-1**). The urticarial rash was disappeared after administration of canakinumab (**a-2** and **b-2**)

A 43-year-old female was referred to our department for evaluation of recurrent febrile attack for 3–4 days, leg pain, and urticarial eruption. No family history of recurrent febrile attack was reported. Three years before the visit, a weekly febrile attack of > 39 °C occurred that lasted 3–4 days. Her symptoms started with urticarial rash in the trunk and legs associated with high fever and progressing leg pains. The urticarial rash initially occurred once every 2 weeks but gradually increased in frequencies. Febrile attacks were accompanied with coxalgia and urticarial eruption not resolved after the fever declined. On physical examination, her blood pressure was 120/72 mmHg, pulse rate was 92 beats/min and respiratory rate 24/min. Chest and abdominal examinations revealed no findings; however, a tenderness was observed in the left hip joint. The temperature at the first visit was 37.1 °C; however, she presented with febrile attack (> 39 °C) 10 days before the first visit. The urticarial rash appeared over the entire body, including the trunk and both upper and lower extremities (Fig. 1). It had aggravated by coldness and accompanied by arthralgia and bone pain of both femurs. The febrile attack lasted for 3–4 days, whereas urticarial rash did not resolve completely. Laboratory findings showed leukocytosis (11,900/μl) and elevated C-reactive protein levels (6.18 mg/dl) and serum amyloid A (SAA, 32.5 μg/ml). Markers for autoimmune diseases, including an anti-nuclear antibody and anti-neutrophil cytoplasmic antibody were negative. There was no M protein in immunoelectrophoresis. Liver and renal functions were negative. Serological testing was negative for HBV, HCV, and Syphilis. Neither cytomegalovirus antigenemia nor EBV or Parbovirus B19 DNA were detected. Interferon γ release assay for *Mycobacterium tuberculosis* infection (T-SPOT) and B-D-glucan tests were both negative. Although one of the diagnostic criteria for SchS requires findings of monoclonal gammopathy, serum immunoelectrophoresis showed no evidence of monoclonal gammopathy. In addition, bone marrow aspirates showed no abnormality.

**Fig. 2:**
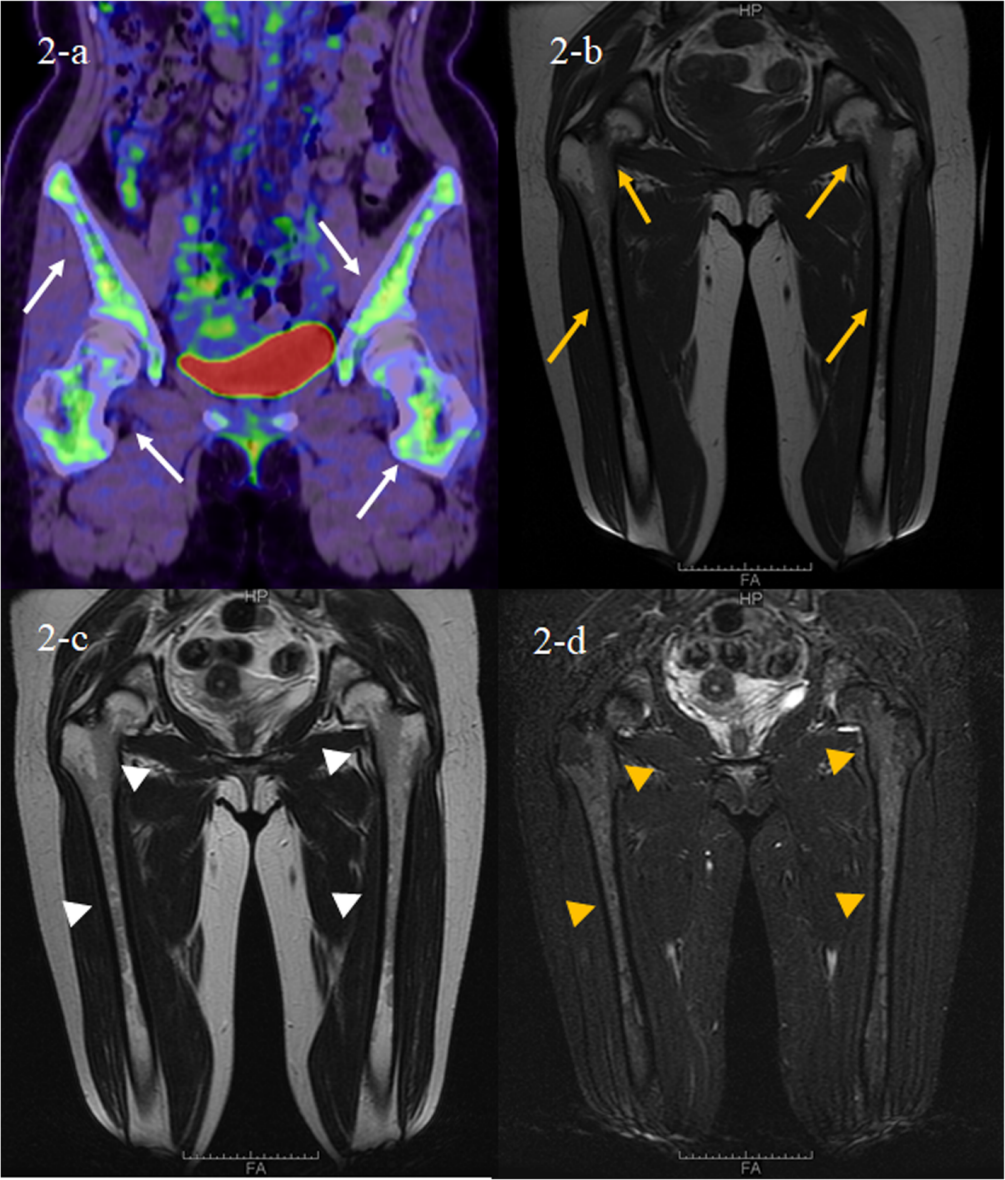
FDG-PET/CT and MRI findings. The FDC-PET/CT findings of femurs demonstrate increased ^18^FDG uptake in the bone marrow at the pelvis and femurs (**a**, white arrows). The MRI findings of femurs demonstrates low signal intensity on the T1-weighted image (**b**, yellow arrows) and T2-weighte image (**c**, white arrowheads) whereas high signal intensity on the STIR image (**d**, yellow arrowheads). ^18^FDG = fluorine-18-fluro-deoxyglucose, PET/CT = positron emission tomography/computed tomography, MRI = magnetic resonance imaging, STIR = short T1 inversion recovery

**Fig. 3:**
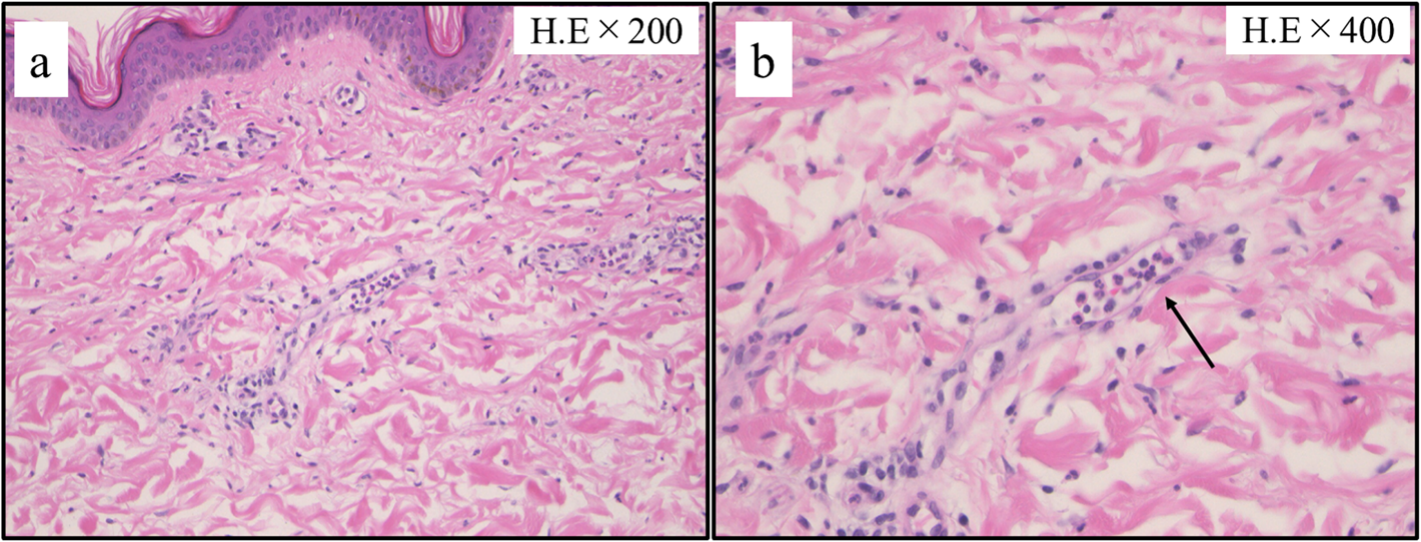
Histopathological findings of skin biopsy specimen. The pathological findings of skin biopsy. **a** Mild infiltration of lymphocytes, eosinophils, and neutrophils around blood vessels in the dermis. **b** Netrophils and eosinophils in blood vessels (arrow)

PET/CT showed the diffuse fluorodeoxyglucose (FDG) uptake in the bone marrow of both femoral and pelvic bones (Fig. 2a). MRI of both femurs (Fig. 2c-d) show diffusely abnormal signal in the medullar bone of both femoral bones. Histological findings of the biopsied urticarial lesions showed a neutrophilic urticarial dermatosis demonstrating perivascular and interstitial neutrophilic infiltrations without leukocytoclastic vasculitis (Fig. 3). Based on the urticarial rash, recurrent fever, abnormal bone remodeling with bone pain, and elevated acute phase reactants, SchS was suspected, despite the absence of monoclonal gammopathy. The patient had been treated with high-dose prednisolon, antihistamines, omarizumab, and cyclosporine A; however, she was unresponsive to these various treatments. The observation that she did not respond to any conventional medications indicated the possibility of autoinflammatory diseases. Therefore, written informed consent for gene analysis for autoinflamatory genes was obtained from the patient, and the ethical approval for the study was obtained from the Fukushima Medical University Ethics Committee for this retrospective study (No 2019–188) and in accordance with the Declaration of Helsinki. In order to exclude the mutations in the genes of autoinflammatory diseases, the genetic screening was performed in genomic DNA samples from the patient whole blood under analysis by next-generation sequencing. A panel was created to identify possibly disease-causing mutations in 14 autoinflammation/immune-related genes (*MEFV, TNFRSF1A,NLRP3,NLRP12,VK,PLCG2,NOD2,TMEM173,PSMB8,PSMA3,PSMB4,PSMB9,POMP,NLRC4*). However, neither mutation nor rare variant was found in these genes (data not shown). Despite the lack of diagnostic criteria for the typical SchS (absence of monoclonal gammopathy) and CAPS (absence of NLRP3 mutation or somatic mosaicism), we did not exclude the possibility of antoinflammatory diseases including SchS. Neutrophilic urticarial with systemic inflammation (NUSI) characterized by urticarial skin lesions with neutrophilic infiltration likely mediate by IL-1 [7]. The differential diagnosis of NUSI should be considered in this case with antihistamine or immunosuppressant-resistant urticaria with systemic inflammation [7]. Exclusion of known inflammatory diseases is necessary for the diagnosis of NUSI [7]. Although inflammatory arthritis could be associated with NUSI [7], bone pain with bone remodeling, one of the clinical findings of SchS, may not be present with NUSI. The lack of monoclonal gammopathy does not necessarily deny SchS because monoclonal gammopathy may not be present at disease onset [8]. She fulfilled the diagnostic criteria for SchS except monoclonal gammopathy.

After these extensive diagnostic workouts, the SchS diagnosis was suspected by bone pain with compatible imaging findings.

Recent guideline suggests a diagnosis of IL-1-neduated autoinflammtory disease such a situation [9]. She suffered from sustained recurrent fever, urticarial rash, and bone pain with the elevated levels of acute phase reactants for more than 3 years. The association of AA amyloidosis was reported in untreated patients with SchS [6] sustaining elevations of acute phase reactant, SAA, contribute to the AA amyloidosis development as a precursor protein for AA amyloid [10].

Therefore, Il-1β monoclonal antibody, canakinumab (Iralis^<^, Novartis) 150 mg was subcutaneously injected with a 4-week interval. Skin rash and bone pain were resolved within 2 days of the first injection. Inflammatory markers, such as CRP and SAA, were normalized after 7 days from the first injection. The patient noticed significant improvement and recurrent febrile attack was completely disappeared.

## Discussion and conclusion

Schnitzler syndrome is a rare autoinflammatory disease with typical skin manifestations [1]. The presence of the typical skin lesion of an urticarial dermatosis and monoclonal gammaopathy by IgM are essential for the disease diagnosis [11]. Our case presented with SchS resembling urticarial skin lesions characterized by perivascular and interstitial neutrophilic infiltrations without vasculitis histologically. Recent imaging study suggested the abnormal bone remodeling involving of long bones in SchS [12]. In our case, abnormal FDG uptake in both proximal femurs in PET/CT and high signals in MRI examination suggesting the high bone remodeling on bone pain lesions. Despite these clinical manifestations resembling SchS, monoclonal gammaopathy was not observed in the present case.

Although monoclonal gammopathy is regarded as a major diagnostic criterion for SchS, the nature of this association remains unclear. In most patients, monoclonal IgM gammopathy without lymphoproliferative disease is present; however, why monoclonal gammopathy is accompanied with SchS remains unknown [13]. A few cases have shown that monoclonal is not detected in the early stage of this syndrome [14]. However, monoclonal gammopathy has been reported to be still absent during the entire disease course [15]. The presence of monoclonal IgM is a mandatory criterion for diagnosing Schnitzler’s syndrome [3]; however, not all patients had monoclonal IgM at the initiation stage of SchS [4]. It was also reported that monoclonal gammopathy may not be present at the disease onset and may present later in the course [8]. Ricardo et al. described a case-based review about SchS without monoclonal gammopathy, in which two out of nine patients developed IgM k chain monoclonal gammopathy within follow up period [16]. The present patient did not present with IgM gammopathy during the follow-up period in which clinical manifestation of SchS was completely resolved by canakinumab treatment.

The role of the paraproteinemia in SchS remains unknown, and the role of paraproteinemia in initiating skin inflammation in SchS has not yet been demonstrated [17]. Moreover, whether the paraproteinemia is an inducer of IL-1 β-mediated autoinflammtion or results from sustained autoinflammation remains unclear [18].

Considering the recurrent febrile attack accompanied with the cutaneous urticarial manifestation observed in the present case could be attributable to other autoinflammatory diseases presenting refractory urticaria, such as cryopyrin-associated periodic syndrome (CAPS) [19]. In CAPS, either germline mutations or somatic mosaicism in the NLRP3 resulted in a spontaneous production of IL-1β [20]. A recent study identified somatic NLRP3 mosaicism in a patient with SchS [21]. These NLRP3 mutations may contribute to the IL-1β induction, resulting in skin inflammation or high fever in autoimflammatory disorder including SchS. A few cases of severe clinical phenotype of SchS have been described with proven NLRP3 gene mutations [22]. However, such mutations are not always present in SchS. NLRP3 mosaicism can be detected by next-generation sequencing [23]; however, we could not completely rule out NLRP3 mosaicism.

Despite its rarity, early diagnosis of SchS is needed, since SchS could be complicated with AA amyloidosis, due to the sustained inflammation [6]. Treatment of SchS remains difficult and unsatisfactory [2]. In particular, conventional medications, such as steroid and immunosuppressant, seem to be ineffective against autoinflammatory conditions involved in the chronic urticaria with systemic inflammation in SchS [24]. IL-1 blocking agents successfully ameliorate the urticarial rash and periodic fever and elevated SAA levels [25, 26], which may eliminate the risk for a serious complication, AA amyloidosis. IL-1 antagonist therapy may efficiently prevent such serious complication [24]. In the present case, immunosuppressive therapies including corticosteroid, omarizumab, or cydosporin A, were infective at all. However, IL-1 blocking therapy using canakinumab dramatically eliminate the clinical manifestations, such as febrile attack, urticarial rash, bone pain, and elevated CRP, or SAA levels.

These findings suggest that IL-1-mediated autoinflammatory processes are involved in the pathophysiology of various clinical manifestations seem in the present case. Although the typical form of SchS is associated with monoclonal gammopathy as a diagnostic criterion, the present case report suggests that monoclonal gammopathy can be absent in patients with refractory urticaria having SchS resembling symptoms, in whom IL-1 blocking therapy seems to be effective.

## Data Availability

Please contact author for data requests.

## Abbreviations

CRP: C-reactive protein
CAPS: Cryopyrin-associated periodic syndrome
FDG: Fluorodeoxyglucose
IL-1β: Interleukin-1 beta
NLRP3: NLR family pyrin domain containing 3
PET: Positron emission tomography
SAA: Serum amyloid A
SchS: Schnitzler’s syndrome
